# LOU/c/jall rat as a model of resilience in the context of streptozotocin-induced cognitive impairment

**DOI:** 10.3389/fnagi.2025.1666397

**Published:** 2025-10-16

**Authors:** Lucas Gephine, Sophie Corvaisier, Benoît Bernay, Thomas Freret, Marianne Leger

**Affiliations:** ^1^Normandie Univ, UNICAEN, INSERM, COMETE, CYCERON, CHU Caen, Caen, France; ^2^Plateforme Proteogen SFR ICORE 4206, UNICAEN, Caen, France

**Keywords:** cognitive resilience, Alzheimer’s disease, preclinical models, Lou/c/jall rat, memory, behavior, proteomic

## Abstract

**Introduction:**

The concept of cognitive resilience (CR) has emerged to explain the lack of correlation between the extent of brain lesions and the severity of cognitive symptoms in Alzheimer’s disease (AD), but the underlying mechanisms remain poorly understood.

**Methods:**

To investigate this, we developed a preclinical model of CR using a sporadic model of AD in a specific rat strain named LOU/c/jall described as a successful aging model. LOU/c/jall and Wistar control rats were bilaterally injected with streptozotocin (STZ; 3 mg/kg) or a vehicle solution into the cerebral ventricles. Cognitive performance and neuropathological examinations were evaluated 1 month after surgery.

**Results:**

Our results showed that STZ-injected Wistar exhibited greater cognitive deficits than LOU/c/jall, despite similar brain alterations, revealing for the first time CR in the LOU/c/jall strain. Proteomic analysis identified differentially expressed proteins involved in the AD pathway between the two strains.

**Discussion:**

Understanding the role of these proteins in AD could improve our understanding of the brain mechanisms underlying CR and guide the development of more targeted therapeutic strategies.

## 1 Introduction

The concept of cognitive resilience (CR) refers to the phenomenon in which individuals show no cognitive impairment despite the presence of neurobiological hallmarks of Alzheimer’s disease (AD) (Negash et al., 2013). Indeed, an increasing number of studies have demonstrated that a significant proportion of subjects with cerebral amyloid deposits and tau pathology remained individuals living without dementia over the course of aging, thereby demonstrating CR to the pathology (Negash et al., 2013). The result is a high degree of inter-individual cognitive variability between AD patients. Such heterogeneity presents a major challenge for the development of effective therapeutic strategies (Perneczky et al., 2019; Stern et al., 2023). Studying CR in clinical practice, however, is constrained by several limitations, including limited access to post-mortem samples and time constraints imposed by the progressive nature of the disease, which complicate longitudinal follow-up. In this context, animal models represent an invaluable resource for advancing our understanding of CR and its underlying mechanisms.

In the context of AD, two main approaches are employed to identify resilient animals. The first approach utilizes the enriched environment (EE) to model CR. Indeed, a slowdown in the spread of the disease was observed in rodents placed in EE (Lazarov et al., 2005), associated with a maintenance of cognitive performances (Verret et al., 2013). EE promotes social interaction, cognitive stimulation, novelty and exercise that contribute to these beneficial effects. These behavioral observations are linked to enhanced hippocampal neurogenesis driven by EE (Salmin et al., 2017) and by the regulation of the balance between BDNF and its precursor, pro-BDNF. While BDNF promotes hippocampal plasticity and improves cognitive performance in AD (de Pins et al., 2019; Hsiao et al., 2014), pro-BDNF is associated with neurotoxic signaling in AD (Fleitas et al., 2018). The second approach involved identifying resilient animals within a given population. For instance, cohorts of Tg2576 mice, a well-established model of AD, can be used to study CR. By aging these animals, resilient and non-resilient subpopulations can be identified. While non-resilient aged Tg2576 mice exhibit impaired spatial memory performance in the Morris water maze (MWM) test as early as 16 months of age, resilient mice maintain performance comparable to that of age-matched control mice (Pérez-González et al., 2020, 2021), providing a suitable tool to investigate the underlying mechanisms of CR. Resilient Tg2576 mice showed increased levels of proteins involved in different processes such as synaptic plasticity and integrity or reduced neuroinflammation (Pérez-González et al., 2020, 2021). Besides, these mice showed preserved dendritic spine plasticity and synaptic machinery in the hippocampus, which would play a crucial role in the mechanisms of CR (Pérez-González et al., 2020, 2021). Other studies have used contextual fear conditioning to identify resilient 5xFAD mice, detected at 8 months of age. Researchers have demonstrated that resilient 5xFAD mice differentially express more than 100 hippocampal proteins compared to their non-resilient counterparts, including proteins involved in neuronal excitability and synaptic plasticity (Neuner et al., 2017).

Overall, the majority of preclinical studies on AD are based on genetic models that represent only 1% of all AD cases. These models exacerbate the amyloid pathology and do not fully reflect the complex pathophysiology of AD (Xu et al., 2023). In addition, the identification of resilient animals within such models requires several months and is currently based solely on prediction, since it is not possible to predict in advance the number of animals that will be resilient to a disease within a cohort. It may therefore be advantageous to use sporadic AD models to more closely reflect clinical reality, combined with a characterized model of CR in order to limit the drawbacks of these studies linked to the assumption of the appearance of CR.

One of the most commonly used sporadic models is the intracerebroventricular (ICV) administration of streptozotocin (STZ). Originally developed in Wistar (WIS) rats, a dose of 3 mg/kg of STZ has been shown to disrupt the brain glucose metabolism (Mayer et al., 1989). One month after STZ administration, hallmarks of AD, including β-amyloid accumulation, phosphorylated tau protein, and neuroinflammation were observed in both the hippocampus and cortical regions (Moreira-Silva et al., 2019). These cerebral changes were associated with significant behavioral impairments. Enhanced anxiety-like behaviour has been observed between 2 and 4 weeks post-injection, as assessed using the elevated plus maze test (Amani et al., 2019; Mehla et al., 2013). Furthermore, deficits in recognition memory (Moreira-Silva et al., 2018), spatial memory (Amani et al., 2019; Tiwari et al., 2021), as well as working memory performance have been reported using the novel object recognition test (NORT), the MWM, and spontaneous alternation in Y-maze test (Amani et al., 2019), respectively. Despite its relevance in mimicking AD-like features, this sporadic model has not yet been used to investigate CR.

Genetically derived from WIS rats, LOU/c/jall (LOU) rats have been described as a model of successful aging. Subjecting themselves spontaneously to caloric restriction, LOU rats exhibit low levels of body fat throughout life, which may explain their longer median lifespan than WIS rats (Alliot et al., 2002). Regarding behavior, reduced anxiety, increased activity, and preserved memory performance during aging have been observed in this strain (Alliot et al., 2002; Kollen et al., 2010; Ménard et al., 2014). Genetic analysis has identified a set of genes in the hippocampus and in the frontal cortex that may be associated with the preservation of the cognitive abilities during aging (Paban et al., 2013). These findings led us to hypothesize that the LOU rat may serve as a model of CR in the context of AD.

Therefore, the aim of our study was to model the heterogeneity of CR by combining a model of successful aging with a rat model of sporadic AD. Using a behavioural and biochemical approach, we hypothesized that LOU rats would demonstrate CR to the STZ treatment compared to WIS rats. Using proteomic analysis, we sought to identify proteins of interest potentially involved in this CR observed in the LOU strain. This approach may facilitate the elucidation of the neuroprotective mechanisms underlying the CR, thereby informing future therapeutic strategies.

## 2 Materials and methods

### 2.1 Animals

All experiments were performed on 2 months old male WIS rats (*n* = 38; “WIS” group; Janvier Labs, France) and age-matched LOU rats (*n* = 37; “LOU” group; CURB, Caen, France). The rats were housed in transparent polycarbonate cages manufactured by Tecniplast^®^ (598 x 380 x 200 cm, 3 rats per cage; containing nesting material and cardboard tube) in a controlled environment (22 ± 2 °C, 55 ± 10% humidity) under a normal 12:12h light/dark cycle (light on at 7:30 AM). Food and water were available *ad libitum*. Following recommendations from the CENOMEXA ethics committee, the number of subjects required was calculated based on data published by our team in a behavioral test assessing working memory in WIS rats (Paban et al., 2013). All the experiments were carried out in accordance with the national and European regulations concerning animal experimentation (EU directive N°2010/63; project authorisation number APAFIS#38885).

### 2.2 Experimental design

**FIGURE 1:**
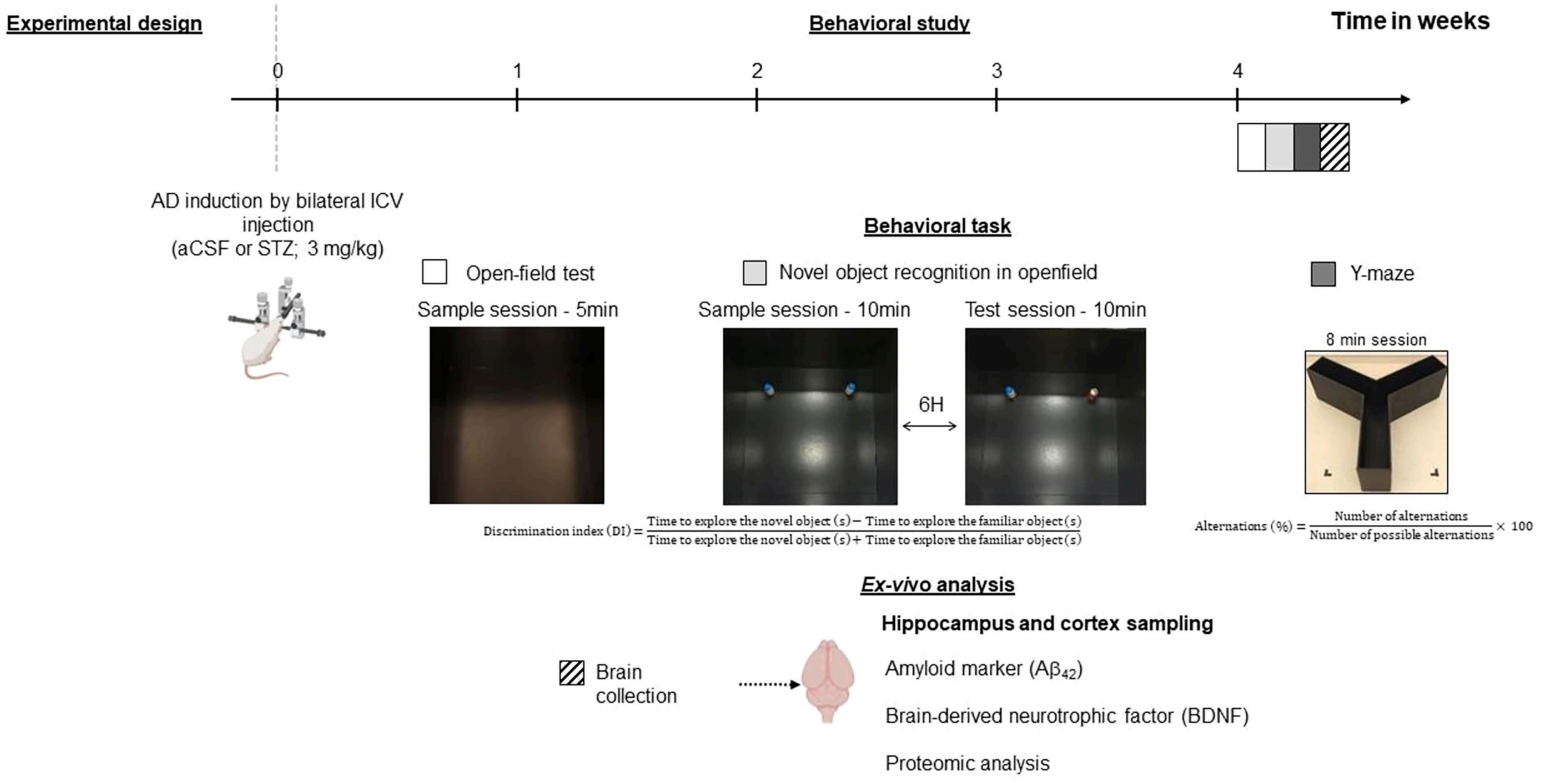
Experimental design. Two-month-old male Wistar (WIS) and LOU/c/jall (LOU) rats were injected with artificial cerebrospinal fluid (aCSF) or 3mg/kg streptozotocin (STZ) into the lateral ventricles of the brain. A behavioural study started 1 month later and was followed by post-mortem cerebral analyses. Created with BioRender.com.

Upon their arrival, the rats were first acclimated to the animal facilities for 1 week and handled on a daily basis before undergoing sporadic AD model surgery. WIS and LOU rats were randomly assigned to either the STZ group or the artificial cerebrospinal fluid (aCSF) group (Figure 1). Behavioral study and brain collection was performed 1 month after surgery (*n* = 19 WIS-aCSF; *n* = 19 WIS-STZ; *n* = 19 LOU-aCSF; *n* = 18 LOU-STZ).

### 2.3 Surgery

The surgical procedure was conducted under deep analgesia (buprenorphine; 0.025 mg/kg; subcutaneous route; 30 min prior to surgery) and anesthesia (induction: 5% isoflurane for induction and 2.5% isoflurane for maintenance in a 30% O_2_, 70% N_2_O mixture) in a stereotaxic frame (World Precision Instruments^®^). The rat was placed on a heating pad (rectal temperature was maintained at 37°C; TemSega^®^) and its ears and eyes were protected with anesthetic cream (lidocaine/prilocaine 5%, Zentiva^®^) and moisturizing gel (Lacryvisc^®^), respectively. The incision site was first shaved and disinfected (Betadine skin 10%). A longitudinal incision was then made to expose the skull. Once the bregma was identified, 0.5 mm diameter cranial perforations were made bilaterally using a high-speed drill (Phymep^®^) to target the lateral ventricles (AP = −0.8 mm, DV = −3.6 mm dorsoventrally, ML = +/−1.5 mm from the bregma) (Paxinos and Watson, 2006). The solutions [aCSF or STZ 3 mg/kg (Moreira-Silva et al., 2019)], were loaded into a cannula connected to a Hamilton syringe (VWR International^®^; 25 μL). STZ solutions (Sigma-Aldrich; CAS: 18883-66-4; Lot: 102471644; powder stored at −20° C) were freshly prepared each day and kept at 4° C prior to injection. The solutions were injected at a perfusion rate of 1 μL/min (5 μl per ventricle). The cannulas were maintained 2 min in position after the end of the injections and were then slowly removed to prevent any reflux of the solutions. The rats were subsequently sutured with Vicryl 4.0 sutures (Ethicon^®^) and placed in an individual cage under heated light until they awoke, at which point they were returned to their original housing cage. A second injection of analgesic (buprenorphine, 0.025 mg/kg subcutaneous route) was administrated 6 h after the surgical procedure.

### 2.4 Behavioral experiments

#### 2.4.1 Openfield test

The spontaneous exploration and anxiety-like behavior of animals were assessed in an OF apparatus (100 × 100 × 60 cm) made of wood and painted black. Each rat was placed in the OF and allowed to explore freely for 5 min. Using Noldus Ethovision XT 17^®^ software, the area was virtually delimited into a central zone (70 × 70 cm) and a peripheral zone. The total distance covered in the device, as well as the times and distances covered in the central and peripheral zones, were collected. Animals spending more time in the central zone of the open field were considered less anxious.

#### 2.4.2 Novel object recognition test (NORT)

The recognition memory of animals was evaluated in the same OF arena previously described. On day 1, each rat was first familiarized to the OF for 10 min (2 sessions/day – the first session was used to assess spontaneous exploration and anxiety-like behavior). On day 2, each rat was exposed to two distinct 10 min sessions, with a 6 h inter-session interval during which the rat returned to its home cage. During the sample session, each rat was allowed to freely explore 2 identical objects placed 55 cm apart, in close proximity to the corners. During the test session, the rat was allowed to freely explore a copy of one of the previous objects (the familiar object) and a new object (the novel object). The position of the objects (left or right) and their nature (coke can or milk bottle) were randomized. Between each session, the objects and apparatus were carefully cleaned with 70° ethanol to remove olfactory cues.

The time spent to explore each object (i.e., defined by active sniffing of the object at a distance below 2 cm) during each session was manually measured. The exploration time of the novel object was then expressed as the discrimination index:


Discrimination⁢index⁢(DI)=

$$\frac{\mathrm{Time}\,\mathrm{to}\,\mathrm{explore}\,\mathrm{the}\,\mathrm{novel}\,\mathrm{object}\,\left(s\right)-\mathrm{Time}\,\mathrm{to}\,\mathrm{explore}\,\mathrm{the}\,\mathrm{familiar}\,\mathrm{object}\,\left(s\right)}{\mathrm{Time}\,\mathrm{to}\,\mathrm{explore}\,\mathrm{the}\,\mathrm{novel}\,\mathrm{object}\,\left(s\right)+\mathrm{Time}\,\mathrm{to}\,\mathrm{explore}\,\mathrm{the}\,\mathrm{familiar}\,\mathrm{object}\,\left(s\right)}$$

A discrimination index significantly higher than 0 (theoretical value linked to chance) was interpreted as reflecting intact recognition memory performance.

#### 2.4.3 Spontaneous alternation test

The spatial working memory was assessed as described before (Paban et al., 2013), in a three-arm maze (50 x 15 x 32 cm each; 60lux) made of wood and painted in black. Each rat was placed in the starting arm, with the head facing the wall, and was allowed to freely explore the maze during 8min. The number of alternations and the order of arm entries were collected and used to determine the percentage of alternation:


$$\mathrm{Alternations}\left(\%\right)=\frac{\mathrm{Number}\,\mathrm{of}\,\mathrm{alternations}}{\mathrm{Number}\,\mathrm{of}\,\mathrm{possible}\,\mathrm{alternations}}\times 100$$

A percentage of alternation that was significantly higher than 50% (theoretical value related to chance) was interpreted as reflecting intact working memory performance.

#### 2.4.4 Tissue collection and samples preparation

Following the administration of an anesthetic overdose (isoflurane 5% in O_2_, 70% N_2_O mixture), the rats were dislocated and decapitated. The brain was extracted and the cortex and hippocampus were isolated. The tissues were frozen in liquid nitrogen and stored at −80° C until biochemical analysis. The hippocampus and cortex were homogenized with a 20 mM Tris-HCL lysis buffer (pH = 7.5) containing 150 mM NaCl and a cocktail of protease and phosphatase inhibitors (cOmplete Mini and PhosSTOP, respectively; Roche^®^). Following a 30 min incubation period at 4° C, the samples were centrifuged (4° C, 10 000 RPM, 20 min) to separate the supernatant, which was then stored at −80° C until analysis.

#### 2.4.5 Biochemical analysis

The concentration of amyloid (Aβ_40_, Aβ_42_) was quantified using the MSD V-plex (K15199E) Aβ peptide panel kit (Meso Scale Diagnostic). Brain derived neurotrophic factor (BDNF) concentration was determined using HTRF BDNF kit (PerkinElmer, 62BDNFPEG). The results were normalized to the total protein amount. All ELISAs were conducted with the instructions provided by the manufacturer.

#### 2.4.6 Proteomic analysis

Five μg of each protein extract were prepared using a modified Gel-aided sample preparation protocol (Fischer and Kessler, 2015). Samples were digested with trypsin/Lys-C overnight at 37° C. For nano-liquid chromatography fragmentation, protein or peptide samples were first desalted and concentrated onto a μC18 Omix (Agilent) before analysis.

The chromatography step was performed on a NanoElute (Bruker Daltonics) ultra-high-pressure nano flow chromatography system. Approximatively 100 ng of each peptide sample were concentrated onto a C18 pepmap 100 (5 mm x 300 μm i.d.) precolumn (Thermo Scientific) and separated at 50° C onto a reversed phase Reprosil column (25cm x 75 μm i.d.) packed with 1.6 μm C18 coated porous silica beads (Ionopticks). Mobile phases consisted of 0.1% formic acid, 99.9% water (v/v) (A) and 0.1% formic acid in 99.9% acetonitrileacetr (v/v) (B). The nanoflow rate was set at 250 nl/min, and the gradient profile was as follows: from 2 to 30% B within 70 min, followed by an increase to 37% B within 5 min and further to 85% within 5 min and reequilibration.

Mass spectrometry experiments were carried out on a trapped ion mobility spectrometry-time of flight pro mass spectrometer (Bruker Daltonics) with a modified nano electrospray ion source (CaptiveSpray, Bruker Daltonics). A 1400 spray voltage with a capillary temperature of 180° C was typically employed for ionizing. MS spectra were acquired in the positive mode in the mass range from 100 to 1700 m/z and 0.60 to 1.60 1/k0 window. In the experiments described here, the mass spectrometer was operated in parallel accumulation serial fragmentation with data independent acquisition mode with exclusion of single charged peptides. The data independent acquisition scheme consisted of 16 variable windows ranging from 300 to 1300m/z.

#### 2.4.7 Protein identification

Database searching and label free quantification (using XIC) was performed using a data-independent acquisition by neural networks (version 1.8.2) (Demichev et al., 2020). An updated *Rattus rattus* database was used for library-free search / library generation. For retention time prediction and extraction mass accuracy, we used the default parameter 0.0, which means data-independent acquisition by neural networks performed automatic mass and retention time correction. Top six fragments (ranked by their library intensities) were used for peptide identification and quantification. The false discovery rate was set to 1% at the peptide precursor level. The variable modifications allowed were as follows: Nterm-acetylation and Oxidation. In addition, C-Propionoamide was set as fix modification. “Trypsin/P” was selected. Data were filtering according to a false discovery rate of 1%. Cross-run normalisation was performed using retention time-dependent.

#### 2.4.8 Identification of differentially expressed proteins

To quantify the relative levels of protein abundance between different groups, data from data-independent acquisition by neural networks were then analyzed using a package for differential enrichment analysis of proteomics from R. Briefly, proteins that are identified in 2 out of 3 replicates of at least one condition were filtered, missing data were imputed from PERSEUS using normal distribution and differential enrichment analysis was based on linear models and empirical Bayes statistic.

#### 2.4.9 Statistical analysis

Statistical analysis and graphical representations were performed using RStudio^®^ and GraphPad Prism^®^, respectively. Prior to analysis, the homogeneity of variances (Levene’s test) and normality of the data (Shapiro-Wilk test) were first assessed. When these two assumptions were met, data were analyzed using parametric statistical tests of analysis of variance (ANOVA, with the strain and treatment as main factors) and represented as mean ± standard error of the mean (SEM). Otherwise, non-parametric equivalent statistical tests were employed (Mann-Whitney tests and two-way non-parametric statistics tests for independent data (R package “permuco” and “Rfit”) followed by Holm test for multiples comparisons) and the data were represented as median ± quartiles. Finally, comparisons with the chance level (50%) were analyzed using univariate *t*-tests or Wilcoxon signed rank tests. For proteomic statistical analyses, a 1.2-fold increase in relative abundance and a 0.05 *p*-value were used to determine enriched proteins.

## 3 Results

### 3.1 LOU rats showed reduced anxiety like behavior compared to WIS rats independently of treatment

**FIGURE 2:**
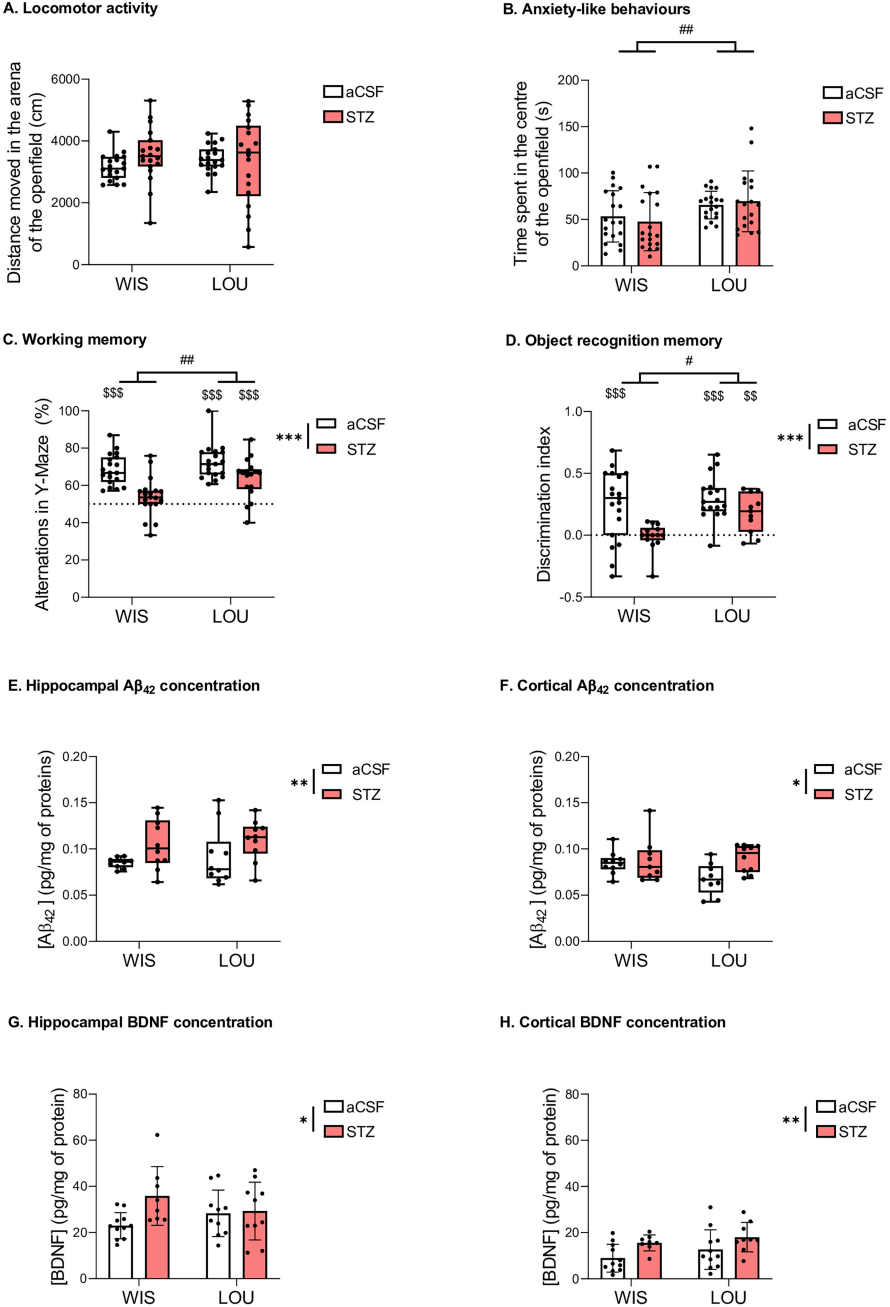
Behavioural and biochemical analysis. **(A, B**) Locomotor activity and anxiety-like behaviour (medians + quartiles; mean ± SEM, *n* = 18–19 per group; 2-way parametric ANOVA: ##*p* < 0.01, significantly higher than the Wistar strain). **(C)** Preserved working memory in LOU-STZ group (medians ± quartiles; *n* = 18–19 per group; 2-way nonparametric ANOVA: ***p < 0.001, significantly lower than the aCSF group; ##*p* < 0.01, significantly higher than the Wistar strain; Univariate *t*-test and Wilcoxon signed rank test: $$$*p* < 0.001, significantly higher than the theorical value of 50%). **(D)** Preserved object recognition memory in LOU-STZ group (medians ± quartiles; *n* = 11–19 per group; 2-way nonparametric ANOVA: ***p < 0.001, significantly lower than the aCSF group; #*p* < 0.05, significantly higher than the Wistar strain; univariate *t*-test and Wilcoxon signed rank test: $$*p* < 0.01, $$$*p* < 0.001, significantly higher than the theorical value of 50%). **(E, F)** Hippocampal and cortical amyloidogenesis in STZ groups (medians ± quartiles; *n* = 14–17 per group; 2-way nonparametric ANOVA: *p < 0.05, **p < 0.01, significantly higher than the aCSF group). **(G, H)** Hippocampal and cortical BDNF increase in STZ groups (mean ± SEM; *n* = 14–17 per group; 2-way parametric ANOVA: *p < 0.05, **p < 0.01, significantly higher than the aCSF group).

The NORT and Y-Maze paradigms rely on exploratory activity, which is influenced by the locomotor activity of each animal. To see whether the treatment could influence this locomotor activity, we evaluated the total distance moved in the arena of the OF in STZ and aCSF-injected control rats. The total distance moved in the arena of the OF did not differ between groups (Figure 2A). However, LOU rats spent more time in the center of the OF than WIS rats (2-way parametric statistics for independent data: strain effect, F_(1,71)_ = 9.64, *p* < 0.01) (Figure 2B), suggesting a reduced anxiety-like behavior.

### 3.2 LOU rats showed preserved working and recognition memory performances compared to WIS rats after STZ treatment

In the Y-maze, LOU rats showed a higher percentage of spontaneous alternation than WIS rats (2-way nonparametric statistics for independent data: strain effect, F = 9.58, *p* < 0.01). In addition, STZ injection induced a decrease in the percentage of alternations (2-way nonparametric statistics for independent data: treatment effect, F = 26.89, *p* < 0.001). Compared to the theoretical value of 50% of performance, STZ induced a significant deficit in alternation percentage in the WIS strain but not in the LOU strain (Wilcoxon signed rank test: *p* < 0.001 for WIS-aCSF, LOU-aCSF, and LOU-STZ groups, compared to 50%, Figure 2C).

During the NORT test session, the discrimination index was higher in LOU rats than in WIS rats (2-way nonparametric statistics for independent data: strain effect, F = 4.16, *p* < 0.05). STZ injection induced an overall decrease in the discrimination index (treatment effect, F = 16.55, *p* < 0.001). Compared to the theoretical value of 0, all groups showed a significant preference for the novel object (Wilcoxon signed rank test: *p* < 0.001 for WIS-aCSF and LOU-aCSF groups, *p* < 0.01 for LOU-STZ group, compared to 0, Figure 2D), except the WIS-STZ group, which showed a recognition memory deficit.

### 3.3 LOU rats showed similar Aβ_42_ and BDNF levels to WIS rats after STZ treatment

Injection of STZ induced an increase in Aβ_42_ production (observed in hippocampus and cortex, 2-way nonparametric statistics for independent data: treatment effect, F = 9.53, *p* < 0.01, and F = 4.91, *p* < 0.05, respectively; Figures 2E, F) and BDNF concentration (2-way ANOVA: treatment effect, F_(1,35)_ = 4.28, *p* < 0.05, and F_(1,35)_ = 8.01, *p* < 0.01, respectively; Figures 2G, H) in both strains. No strain effect was observed.

### 3.4 Proteomic analysis

A proteomic analysis was conducted on hippocampal and cortical samples to examine protein regulation in STZ-injected compared to aCSF-injected rats in each strain. In WIS rats, 8281 protein groups were identified and quantified in the cortex, with 459 (5.5%) showing significant up- or down-regulation. In the hippocampus, 7853 protein groups were identified and quantified, with 375 proteins (4.8%) showing significant changes in regulation. Similarly, in LOU rats, 8294 protein groups were identified and quantified in the cortex, with 398 (4.8%) exhibiting significant up- or down-regulation. In the hippocampus, 898 protein groups (11.8%) were significantly up- or down-regulated among the 7598 protein groups identified and quantified. All the proteins identified and quantified can be find in the Supplementary Data 1.

**FIGURE 3:**
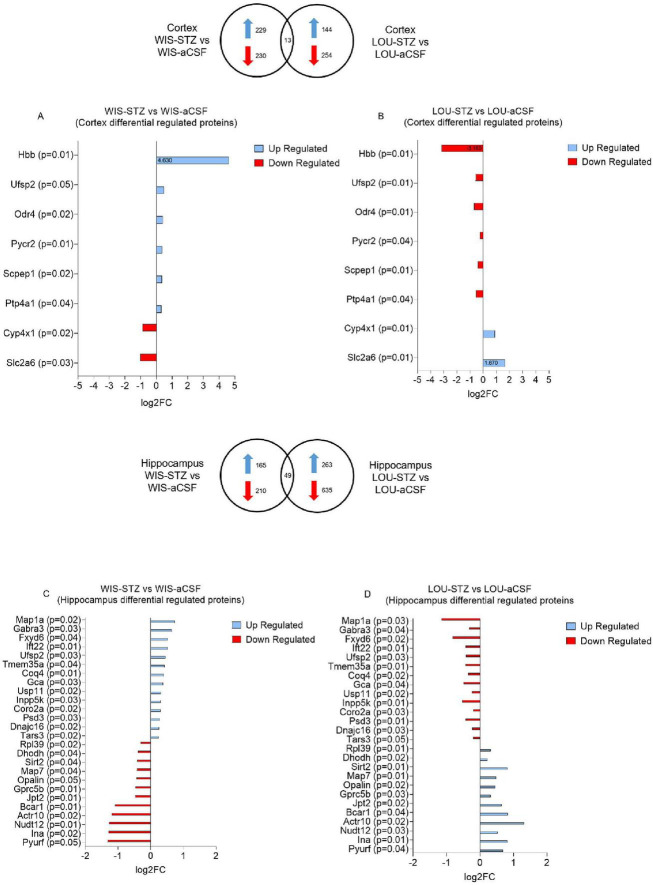
Protein groups differentially up-and down-regulated in WIS and LOU brain samples after STZ treatment. **(A, B)** Differentially regulated proteins in the cortex of WIS-STZ and LOU-STZ compared to WIS- aCSF and LOU-aCSF respectively. **(C, D)** Differentially regulated proteins in the hippocampus of WIS-STZ and LOU-STZ compared to WIS-aCSF and LOU-aCSF respectively. A positive Log2FC indicates up-regulated proteins (blue) while a negative Log2FC indicates down-regulated proteins (red).

In the cortex, 13 proteins common to both strains were identified as being differentially regulated after STZ injection. Notably, 8 of these 13 proteins were oppositely regulated between the two strains. Specifically, Hbb, Ufsp2, Odr4, Pycr2, Scpep1 and Ptp4a1 were up-regulated in the WIS strain, while Cyp4x1 and Slc2a6 were down-regulated in the WIS strain (Figures 3A, B).

In the hippocampus, 49 proteins common to both strains were found to be differentially regulated after STZ injection. Among these, 26 were oppositely regulated between the two strains. Map1a, Gabra3, Fxyd6, Ift22, Ufsp2, Tmem35a, Coq4, Gca, Usp11, Inpp5k, Coro2a, Psd3, Dnajc16 and Tars3 were up-regulated in the WIS strain, while Rpl39, Dhodh, Sirt2, Map7, Opalin, Gprc5b, Jpt2, Bcar1, Actr10, Nudt12, Ina and Pyurf were down-regulated in the WIS strain (Figures 3C, D).

To investigate which of these differentially regulated proteins might be involved in the mechanisms underlying CR, we carried out a literature search specifically on the 34 proteins that were differentially regulated after STZ injection. Interestingly, 3 cortical and 10 hippocampal proteins (Tables 1, 2, respectively) have already been studied in the context of AD pathology.

**TABLE 1:** Cortical proteins that were oppositely regulated between the two strains and that have been studied in the context of AD in the literature.

Gene name	Protein	WIS Log2FC	WIS *p*-value	LOU Log2FC	LOU p-value	Links with AD pathology	Method of analysis
Hbb	Globin a4	4.630	0.010	−3.160	0.012	Increased levels in AD patients plasma (Ashraf et al., 2020) Decreased labeling in brain neurons of AD patients (Ferrer et al., 2011)	Mass spectrometry (Ashraf et al., 2020) Immunohistochemistry and Drabkin’s method (Ferrer et al., 2011)
Ufsp2	Ufm1-specific protease 2	0.488	0.046	−0.576	0.006	Decrease in soluble Ufsp2 and increase in insoluble Ufsp2 in frontal and temporal cortex of AD patients (Yan et al., 2024)	MSD ELISA (Yan et al., 2024)
Ptp4a1	Protein tyrosine phosphatase type IVA 1	0.318	0.040	−0.563	0.040	Elevated serum antibody responses to AD patients, potential biomarker (Wang Y. et al., 2023)	Antibody microarray (Wang Y. et al., 2023)

A positive Log2FC indicates up-regulation while a negative Log2FC indicates down-regulation of the protein. Proteins were classified according to their log2FC values in WIS strain.

**TABLE 2:** Hippocampal proteins that were oppositely regulated between the two strains and that have been studied in the context of AD in the literature.

Gene name	Protein	WIS Log2FC	WIS *p*-value	LOU Log2FC	LOU *p*-value	Links with AD pathology	Method of analysis
Map1a	Microtubule-associated protein 1A	0.739	0.024	−1.150	2.9E-03	Decreased Map1a phosphorylation in the hippocampus of AD rat model. (Cai et al., 2023)Down-regulation in primary cultures of cortical neurons exposed to Aβ_42_ in rats (Clemmensen et al., 2012) and mouse models of AD (Fifre et al., 2006)	Mass spectrometry and quantitative phosphoproteomics (Cai et al., 2023) Immunohistochemistry (Clemmensen et al., 2012; Fifre et al., 2006)
Gabra3	Gamma-aminobutyric acid receptor subunit alpha-3	0.645	0.033	−0.328	4.4E-02	Down-regulation in the middle temporal gyrus of AD patients (Govindpani et al., 2020)	NanoString nCounter and immunohistochemistry (Govindpani et al., 2020)
Fxyd6	FXYD domain-containing ion transport regulator 6	0.524	0.039	−0.822	2.4E-02	Decrease in the brain of Tg2576 mouse model of AD and in hippocampus of AD patients (George et al., 2010)	Real-time PCR gene expression (George et al., 2010)
Ufsp2	Ufm1-specific protease 2	0.450	0.025	−0.418	3.1E-02	Decrease in soluble Ufsp2 and increase in insoluble Ufsp2 in frontal and temporal cortex of AD patients (Yan et al., 2024)	MSD ELISA (Yan et al., 2024)
Coq4	Ubiquinone biosynthesis protein COQ4 homolog, mitochondrial	0.403	0.010	−0.352	2.4E-02	Coq4 is a functional substitute for Coq10, which is necessary for its synthesis. Intraperitoneal injection of Coq10 enhanced cognition, hippocampal BDNF levels and neurogenesis in rat model of AD induced by STZ (Sheykhhasan et al., 2022)	Behavioral analysis and biochemistry (Sheykhhasan et al.)2022
Usp11	Ubiquitin carboxyl-terminal hydrolase 11	0.318	0.019	−0.244	2.3E-02	Inhibition of Usp11 reduced pTau and and Aβ_42_ plaques in the hippocampus of 5xFAD mice (Guo et al., 2024)	Immunohistochemistry (Guo et al., 2024)
Dnajc16	DnaJ homolog subfamily C member 16	0.262	0.024	−0.236	2.6E-02	Identified among AD-associated genes in a transcriptomic dataset of hippocampal structure in AD patients (Zhang et al., 2015)	Hippocampus microarray expression (Zhang et al., 2015)
Rpl39	Large ribosomal subunit protein eL39	−0.310	0.017	0.311	1.2E-02	Down-regulation in blood of AD patients (Fernández-Martínez et al., 2020)	Multi-tissue RNA signature (Fernández-Martínez et al., 2020)
Dhodh	Dihydroorotate dehydrogenase (quinone), mitochondrial	−0.391	0.037	0.227	2.1E-02	Increased Dhodh/Uck2 ratio in the enthorinal cortex of AD patients (Pesini et al., 2019) Pharmacological inhibition dampens CA1 hyperexcitability under anesthesia in APP/PS1 mice (Zarhin et al., 2022)	qPCR, ELISA (Pesini et al., 2019) Local field potential / Field excitatory postsynaptic potentials (Zarhin et al., 2022)
Sirt2	NAD-dependent protein deacetylase	−0.422	0.038	0.819	8.3E-04	Increase in temporal cortex (Silva et al., 2017), CSF (Gaetani et al., 2021) and plasma (Wongchitrat et al., 2019) of AD patients. Pharmacological inhibition reduces cognitive deficits and promote non amyloidogenic processing of APP in 3 x Tg mice (Bai et al., 2022)	Immunohistochemistry (Silva et al., 2017) multiplex PEA technology (Gaetani et al., 2021) Real-time PCR (Wongchitrat et al., 2019) Behavioral analysis and immunohistochemistry (Bai et al., 2022)

A positive Log2FC indicates up-regulation while negative Log2FC indicates a down-regulation of the protein. Proteins were classified according to their log2FC values in WIS strain.

## 4 Discussion

By combining for the first time a model of sporadic AD with a rat model of successful aging, we demonstrated that the LOU strain exhibited CR in the context of streptozotocin-induced cognitive impairment. Indeed, we showed that the LOU strain maintained cognitive performance after ICV injection of STZ compared to WIS, in the presence of similar brain amyloidogenesis.

Locomotor activity and anxiety-like behavior were assessed by measuring the total distance traveled in the OF and the time spent in the center of the arena. Consistent with the literature using the same paradigm on the WIS strain (Amani et al., 2019; Andrade et al., 2023), STZ injection did not impair locomotor activity or anxiety like behavior. It is worth noting that an increase in anxiety-like behavior following STZ injection has been reported in some studies using the elevated plus maze test (Amani et al., 2019). However, we did not observe such effect in our experiments (Supplementary Figure 1 and Supplementary Data 2). In LOU rats, we observed no changes in locomotor activity or anxiety-like behavior following STZ injection. Regardless of the treatment, LOU rats spent more time in the center of the OF arena than WIS rats, supporting the low anxiety phenotype previously described (Ménard et al., 2014). However, such strain-dependent variation in anxiety-like behavior is unlikely to affect memory performance assessments, as it was found to be independent of STZ treatment.

We observed significantly elevated beta-amyloid peptide concentrations in the hippocampus and cortex of WIS-treated animals, which is consistent with the literature on this model (Choi et al., 2014; Salkovic-Petrisic et al., 2011). Interestingly, STZ treatment induced a similar increase in brain beta-amyloid peptide concentrations in LOU rats, supporting our CR hypothesis in this strain (See also Supplementary Figure 2).

In order to identify resilient subjects, the majority of studies mainly use two behavioral tests which are the MWM and the contextual fear conditioning (Aguado et al., 2024; Neuner et al., 2017; Pérez-González et al., 2020, 2021). Unfortunately, the MWM protocol we used did not allow us to identify resilient animals among those treated with STZ, even if the STZ is known to induce spatial memory impairments in this task (Bavarsad et al., 2020; Bokare et al., 2018; Mehla et al., 2013).Moreover, our research has shown that STZ model was not referenced to induce deficits in the contextual fear conditioning (Moreira-Silva et al., 2018; Qi et al., 2021; Wei et al., 2022). Consequently, we chose to use two other less aversive experimental protocols in which STZ injection was known to induce deficits. Spatial working memory and recognition memory were assessed through the measurement of spontaneous alternation (Y-Maze) and the spontaneous objects exploration (NORT), respectively. For both tasks, our results indicated that the aCSF groups exhibited intact memory performance, as their percentage of alternation and novel object discrimination were significantly higher than the chance level (Grayson et al., 2015; Paban et al., 2013). As anticipated in this AD model (Amani et al., 2019; Moreira-Silva et al., 2019), STZ treatment reduced both working and recognition memory performances in both strains. Nevertheless, the percentage of alternation in the LOU-STZ group remained above the chance level, suggesting some preservation of memory, compared to WIS rats. Taken together, our results from the Y-maze and the NORT suggest that LOU rats displayed better memory preservation than WIS rats following STZ treatment.

Our results therefore showed that cognitive performances were maintained in LOU-treated rat in the presence of neuropathological hallmarks similar to those found in the WIS-treated rat confirming our initial hypothesis that the LOU strain could be a CR strain in the context of cognitive impairment induced by STZ.

One hypothesis we have considered to explain this CR involves the levels of hippocampal and cortical BDNF. This neurotrophine is known to promote immune processes, axonal growth, synaptic and dendritic plasticity, as well as learning and memory (Mu et al., 1999; Neumann et al., 1998). Moreover, one study showed an increase in hippocampal and cortical BDNF concentration in LOU rats compared to WIS, suggesting that this observation could be a hypothesis explaining why the LOU strain is considered as a successful aging strain (Silhol et al., 2008). Relevantly, authors highlighted that the difference between both strains was that during aging WIS strain developed more pro-BDNF form compared to LOU strain that is involved in neurotoxic pathways related to AD (Fleitas et al., 2018). Our findings contrast with the existing literature, showing an increase in BDNF concentration in STZ-treated rats. Indeed, a few studies reported a reduction in hippocampal and cortical BDNF concentrations in STZ-treated WIS rats (Bokare et al., 2018; Tiwari et al., 2021). However, the number of injections and the concentration of STZ injected in rat differed from our study. It would also be relevant in future studies to evaluate the levels of pro- and pre-pro BDNF isoforms in order to assess their distribution following STZ treatment and determine whether there is an increase in the pathogenic pro-BDNF isoform, which is known to be involved in neurotoxic pathways associated with AD. Moreover, unlike Silhol’s study, we did not find an increase in basal BDNF concentration in LOU strain compared to WIS, although levels in WIS rats were similar between our studies. However, some clinical studies have reported an increase in BDNF levels in the cortex and hippocampus of AD patients (Laske et al., 2006), as well as elevated plasma BDNF levels in the early stages of AD (Song et al., 2015). A similar increase in cerebral BDNF levels has been observed in the APP23 mouse model of AD, where BDNF was co-located with amyloid plaques (Burbach et al., 2004). These results suggested that in the context of progressive neurodegeneration, the brain may initiate compensatory functional changes and repair mechanisms, involving the upregulation of specific neurotrophic factors in vulnerable regions such as the hippocampus and cortex (Arendt et al., 1995). Unfortunately, we are not aware of any studies evaluating BDNF concentrations in STZ-injected rats under conditions strictly similar to our own.

In order to explore more hypothesis that could explain this CR observed in LOU rats, we then carried out a proteomic study to identify the different proteins potentially involved. We decided to focus our study on the proteins differentially regulated between the both strains and that have been linked to AD. Proteomic analysis revealed many mains findings. First, there were almost 4 times more differentially regulated proteins between the both strains in the hippocampal region (49 proteins) than in the cortical region (13 proteins). In addition, STZ injection mainly down regulated proteins in both strains. Indeed, the percentages of down-regulated proteins were around 50 and 56% in the cortex of WIS and LOU and around 64% and 71% in the hippocampus of the both strains respectively. Finally, LOU rats exhibited proteins that were differentially regulated compared to WIS rats, suggesting potential candidates underlying the observed CR in this strain.

Among the proteins listed, Ufsp2 protein was the only one to be differentially regulated between the both strains and the both structures (hippocampus and cerebral cortex) of STZ-treated animals. Ufsp2 protein, also known as UFM1-specific peptidase 2, is a cysteine protease involved in the UFMylation pathway. This pathway is crucial for various cellular processes including DNA damage response (Qin et al., 2019), immune response (Zhou et al., 2024) or brain development (Wang X. et al., 2023; Zhou et al., 2024). It has recently been shown that the concentration of soluble Ufsp2 specifically decreases in the temporal cortex of AD patients, while the concentration of insoluble Ufsp2 increases in temporal and frontal cortices (Yan et al., 2024).

Interestingly, our proteomic results, based mainly on the analysis of soluble forms, indicate an up-regulation of the Ufsp2 protein in the cortex and hippocampus of STZ-injected WIS rats, whereas a down-regulation was observed in STZ-injected LOU rats. Considering that some have shown ufsp2 KO neurons to be less sensitive to DNA damage in the context of AD (Yan et al., 2024), the down-regulation of ufsp2 in STZ-injected LOU may therefore reflect a protective factor against AD-like pathology.

Another protein of interest was the Usp11 protein. Located on the X chromosome, this protease involved in cell cycle regulation specifically cleaves ubiquitin, regulating the ubiquitination of associated proteins. Among these proteins, Usp11 is involved in the ubiquitination of Tau protein, inhibiting its degradation and exacerbating its accumulation in the formation of neurofibrillary tangles in the context of AD (Guo et al., 2024). Inhibition of Usp11 activity, whether genetic (Yan et al., 2022) or drug-induced (Guo et al., 2024), led to a decrease in pTau, Aβ_42_ burden and an increase in synaptic plasticity and spatial memory in transgenic mouse models of AD, particularly in females. To our knowledge, Usp11 protein expression has never been studied in WIS strain, particularly in the context of AD. Our results, demonstrating an up-regulation in WIS-STZ rats, are in line with the literature showing an improvement of cognitive performances in AD models in which Usp11 protein has been inhibited. Interestingly, our results indicated a down-regulation of Usp11 protein in the hippocampus of STZ-injected LOU rats. Once again, these results are consistent with the maintenance of cognitive performance observed in STZ-injected LOU rats and suggest that this protein may be involved in the CR observed in this strain. With regard to TAU pathology, studies of STZ are more contradictory, as some have shown an increase in TAU genes expression in hippocampus of WIS-treated rats (Rostami et al., 2017) and other strains of rats (Lu et al., 2017; Tiwari et al., 2021), while others have shown a decrease (Moreira-Silva et al., 2018) or no modification of the expression of TAU and p-TAU in hippocampus and cortex (Motzko-Soares et al., 2018). The results of our proteomic analysis showed no variation in the regulation of the neurofibrillary tangle protein in our different groups (see all identified proteins in Supplementary Data 1).

Hemoglobin β (Hbb) undergoes dynamic alterations over the course of AD. In plasma, Ashraf et al. (2020) reported elevated Hbb levels in AD patients, associated with greater amyloid burden, hippocampal atrophy, and cognitive decline, suggesting its potential as a peripheral biomarker. In contrast, post-mortem analyses revealed marked neuronal depletion of hemoglobin subunits (α, β) in cortical and hippocampal neurons containing amyloid-β and tau aggregates, especially at advanced Braak stages (Ferrer et al., 2011). Mechanistic insight from APP/PS1 mice shows that Hbb expression rises with age and amyloid pathology, where it binds amyloid-β via its heme group and promotes plaque formation (Chuang et al., 2012). Collectively, these findings support a biphasic role for Hbb: early upregulation as a compensatory response to hypoxia and metabolic stress, followed by sequestration into amyloid aggregates and neuronal loss in advanced AD. Consistent with these observations, we found that Hbb was upregulated in the cortex of WIS-STZ rats but downregulated in LOU-STZ rats. The upregulation observed in WIS-STZ rats may represent a compensatory response during the early phase of β-amyloid accumulation induced by STZ administration, whereas the downregulation in LOU-STZ rats may reflect strain-specific mechanisms underlying their cognitive resilience.

In addition to the proteins mentioned above, the proteomic study showed a differential regulation of proteins involved in microtubule stability, such as the Map1a protein, which was down-regulated in WIS hippocampus and up-regulated in LOU hippocampus. Similarly, proteins involved in the stability of ribosomal and mitochondrial complexes showed opposite regulation between WIS and LOU strains, with Rpl39 and Dhodh being down-regulated, while Coq4 was up-regulated in WIS hippocampus. It is now clearly established that microtubule degradation occurs in the context of AD, and is partly mediated by Tau pathology (Cai et al., 2023). Other studies have highlighted the role of amyloid pathology in the degradation of the Map1a protein, which is thought to be an early event leading to synaptic dysfunction (Clemmensen et al., 2012; Fifre et al., 2006). In the same way, the dysregulation of both the ribosome and the mitochondrial complexes have been characterized in the context of STZ-injected WIS (da Silva et al., 2024). The overall down-regulation of these proteins in WIS rats following STZ treatment may thus underlie the memory deficits observed.

Taken together, the differential protein expression observed between WIS and LOU rats reinforces the interest in pursuing our efforts to determine whether these proteins are involved in the CR reported here in the LOU strain. In addition to focusing on proteins specifically associated with AD pathology, we have identified other pathways of interest, characterized by differently expressed proteins. This opens up new prospects for the characterisation of proteins involved in CR to AD.

### 4.1 Limits and amelioration perspectives

#### 4.1.1 Proteomic

We chose to focus our study on proteins that are differentially regulated between our both strains and that have already been studied in the context of AD. There is another method of approach that is widely used in literature, which consist of studying the most highly regulated proteins (up and down). This alternative method could reveal other proteins that are more or less highly regulated in the LOU strain and are potential candidates for the CR observed. In addition, the observed up- or downregulation of proteins following STZ administration, such as Ufsp2, will need to be validated through targeted protein quantification by Western blot analyses.

Another limitation is that the proteomic analysis is carried out on cytosolic fractions, which neglects the study of membrane proteins that could potentially be involved in the observed CR. Although the study of membrane fractions is more complex, there are adaptations of the ultra-high pressure nano-flow liquid chromatography protocol for these analyses.

#### 4.1.2 Model

In the present study, CR was investigated in the STZ model (3 mg/kg, single bilateral injection) using 2-month-old male rats, consistent with the majority of STZ-based studies employing male animals between 2 and 3 months of age (Amani et al., 2019; Andrade et al., 2023; Bokare et al., 2018; Correia et al., 2011; Mehla et al., 2013; Moreira-Silva et al., 2018; Motzko-Soares et al., 2018; Rostami et al., 2017). Animals at 2 months of age were selected to ensure a well-controlled assessment of STZ-induced cognitive impairment, as using older rats could confound age-related changes with pathology. This is particularly relevant for LOU rats, which maintain preserved cognitive performance over aging and age differently from other strains (Kollen et al., 2010; Ménard et al., 2014; Paban et al., 2013). The choice of 2-month-old male animals thus allowed us to study STZ effects while minimizing confounding influences of aging. It would also be relevant to carry out a new study including female rats. Indeed, clinical studies have shown that gender has an effect on CR in AD. While in basal conditions, women have a better memory than men, in pathological conditions, it would seem that the decline in cognitive performance is more marked in women than in men, for similar neuropathological conditions (Arenaza-Urquijo et al., 2024).

In order to meet the replication requirements for primary findings, we conducted a complementary study using LOU rats. This study confirmed the preservation of cognitive performance in STZ-animals, thus supporting our initial results. However, the biochemical analyses performed later (3 months) did not reveal any evidence of amyloidogenesis in the STZ-animals, suggesting that the neuropathological effects of STZ may be transient and would require further long-term studies to be fully understood. Further investigations are needed to better validate the model and delineate its pathophysiological profile, particularly the temporal progression of amyloid, tau, and neuroinflammatory changes. A more detailed analysis of amyloid pathology, including the quantification of insoluble Aβ42 aggregates forming plaques, is essential to confirm their presence and clarify their contribution in disease mechanisms. Beyond amyloid, additional markers of AD pathology would deserve to be investigated, including tau pathology, neuroinflammation, and oxidative stress. While these complementary pathways were outside the primary scope of our study on cognitive resilience, their systematic assessment would provide a more comprehensive understanding of the model and bridge the gap between amyloid-centric views and the multifactorial nature of AD pathology.

To our knowledge, this is the first study to demonstrate that LOU rat could be considered as a CR strain in the context of streptozotocin-induced cognitive impairment by the preservation of cognitive performance under conditions of amyloidogenic challenge. The contribution of the proteomic study enabled us to identify various proteins of interest, differentially regulated in the STZ-injected LOU strain compared to the STZ-injected WIS strain, and likely to be involved in CR in the LOU strain.

## Data Availability

The original contributions presented in the study are included in the article/Supplementary material, further inquiries can be directed to the corresponding author/s.
