# Supplementary material for: LOU/c/jall rat as a model of resilience in the context of streptozotocin-induced cognitive impairment
Source: Front Aging Neurosci. 2025 Oct 16;17:1666397. doi: 10.3389/fnagi.2025.1666397 (PMC12571741; doi:10.3389/fnagi.2025.1666397)
Supplement: Supplementary file 2 [file Table_2.docx]

The elevated plus maze consisted of two open arms (50×10×1cm; 170 lux) and two closed arms (50×10×42cm; 15 lux) arranged in a plus shape and connected by a central platform (11×10 cm), elevated 50 cm above the floor. Each rat was placed on the central platform, facing an open arm, and allowed to explore the maze freely for 5 minutes. The time spent in each arm (open or closed) and the number of entries into each were recorded. Anxiety-like behavior was evaluated using two primary measures: the percentage of entries into the open arms and the percentage of time spent in the open arms (Rizzolo et al., 2021). Increased values in either of these parameters were considered indicative of reduced anxiety-like behavior. The total number of arm entries was used as an index of general locomotor activity.

Our findings showed no significant differences between strains or treatments in either the percentage of time spent in the open arms (2-way nonparametric statistics for independent data: no strain effect, F = 0.82, p>0.05; no treatment effect, F = 2.46, p>0.05; **Supplementary Figure S1.A**) or the percentage of entries into the open arms (2-way parametric statistics for independent data: no strain effect, F_(1,58)_ = 0.10, p>0.05; no treatment effect, F_(1,58)_ = 3.24, p>0.05; **Supplementary Figure S1.B).**

**Supplementary Figure S1. Anxiety-like behavior assessed in the Elevated Plus Maze.**
**A.** Percentage of time spent in the open arms (medians ± quartiles; n = 18–19 per group).
**B.** Percentage of entries into the open arm (mean ± SEM; n = 18–19 per group).

Our data showed that STZ injection led to increase the Aβ40 concentration in both hippocampus (2-way nonparametric statistic for independent data: treatment effect, F=12.33, p<0.01, **Supplementary Figure S2.A**) and cortical structures (2-way nonparametric statistic for independent data: treatment effect, F=5.09, p<0.01, **Supplementary Figure S2.B**). Given the increase in Aβ40 and Aβ42, the ratio remained unchanged in both structures (**Supplementary Figure S2.C** and **S2.D**).

**Supplementary Figure S2. Cerebral amyloid levels. A and B. Hippocampal and cortical Aβ40 concentration (medians ± quartiles; n=10 per group; 2-way nonparametric ANOVA: *p<0.05, **p<0.01, significantly higher than the aCSF group). C and D. Hippocampal and cortical Aβ42/ Aβ40 ratio (medians ± quartiles; n=10 per group).**
